# Relationship Between Dietary Nutrient Intake and Autophagy—Related Genes in Obese Humans: A Narrative Review

**DOI:** 10.3390/nu16234003

**Published:** 2024-11-22

**Authors:** Martyna Bednarczyk, Nicola Dąbrowska-Szeja, Dariusz Łętowski, Sylwia Dzięgielewska-Gęsiak, Dariusz Waniczek, Małgorzata Muc-Wierzgoń

**Affiliations:** 1 Department of Cancer Prevention, Faculty of Public Health, Medical University of Silesia in Katowice, 40-055 Katowice, Poland; martyna.bednarczyk@outlook.com (M.B.); nicola.szeja@sum.edu.pl (N.D.-S.); seweryn.letowski@gmail.com (D.Ł.); 2 Department of Internal Diseases Propaedeutics and Emergency Medicine, Faculty of Public Health in Bytom, Medical University of Silesia in Katowice, 40-055 Katowice, Poland; sgesiak@sum.edu.pl; 3 Department of Oncological Surgery, Faculty of Medical Sciences in Zabrze, Medical University of Silesia in Katowice, 40-055 Katowice, Poland; dwaniczek@sum.edu.pl

**Keywords:** nutrients, dietary intake, autophagy, overweight, obesity, humans

## Abstract

Obesity is one of the world’s major public health challenges. Its pathogenesis and comorbid metabolic disorders share common mechanisms, such as mitochondrial or endoplasmic reticulum dysfunction or oxidative stress, gut dysbiosis, chronic inflammation and altered autophagy. Numerous pro-autophagy dietary interventions are being investigated for their potential obesity-preventing or therapeutic effects. We summarize current data on the relationship between autophagy and obesity, and discuss various dietary interventions as regulators of autophagy-related genes in the prevention and ultimate treatment of obesity in humans, as available in scientific databases and published through July 2024. Lifestyle modifications (such as calorie restriction, intermittent fasting, physical exercise), including following a diet rich in flavonoids, antioxidants, specific fatty acids, specific amino acids and others, have shown a beneficial role in the induction of this process. The activation of autophagy through various nutritional interventions tends to elicit a consistent response, characterized by the induction of certain kinases (including AMPK, IKK, JNK1, TAK1, ULK1, and VPS34) or the suppression of others (like mTORC1), the deacetylation of proteins, and the alleviation of inhibitory interactions between BECN1 and members of the Bcl-2 family. Significant health/translational properties of many nutrients (nutraceuticals) can affect chronic disease risk through various mechanisms that include the activation or inhibition of autophagy. The role of nutritional intervention in the regulation of autophagy in obesity and its comorbidities is not yet clear, especially in obese individuals.

## 1. Introduction

Obesity is one of the world’s major public health challenges. Projections indicate that by 2035, approximately 3.3 billion adults will have a high Body Mass Index (BMI > 25), a significant rise from 2.2 billion in 2020. This indicates an increase from 42% of adults in 2020 to more than 54% by 2035 [1]. In Poland, more than 35% of adult men (aged 20 years or older) and more than 25% of adult women will be struggling with obesity in 2035. Also, obesity among children and adolescents will increase year after year.

Based on current trends, it is estimated that by 2035, over 750 million children aged 5 to 19 will be classified as overweight or obese according to BMI. This indicates that approximately 40% of children globally will be impacted, with most of them living in middle-income countries [2].

Obesity is defined as the excessive accumulation or abnormal distribution of body fat (globally, regionally, and in organs as ectopic lipids) that poses health risks [3]. The World Obesity Federation has recognized obesity as a chronic, recurrent and progressive disease [4]. Several factors can play a role in gaining and retaining excess weight. These include: genetics/physiology (for example, metabolism, appetite, satiety and body fat distribution), health inequalities, environmental factors, and commercial determinants (for example, media and advertising, retail environments) [4,5].

Overweight and obesity are the key risk factors for numerous non-communicable diseases (NCDs), such as hypertension, type 2 diabetes mellitus, metabolic syndrome, cardiovascular disease, musculoskeletal disorders and 13 types of cancer. Out of the 41 million adult deaths annually attributed to non-communicable diseases (NCDs), 5 million are due to a high BMI. Almost 4 million of these deaths are directly associated with diabetes, stroke, coronary heart disease, and cancer [1,6,7]. The coexistence of these metabolic disorders impairs liver function, manifesting as Metabolic Dysfunction–Associated Liver Disease (MASLD) [8,9,10].

The pathogenesis of obesity and concomitant metabolic disorders shares common mechanisms such as mitochondrial dysfunction, endoplasmic reticulum or oxidative stress, chronic inflammation, intestinal dysbiosis, and altered autophagocytosis [11].

According to recent studies, it is clear that the common denominator of such metabolic diseases is autophagy—a process that may play an important role in adipose tissue differentiation and function [12,13,14,15,16]. In the initial phases of adipose tissue differentiation and adipogenesis, there is a huge increase in both the quantity of mitochondria and mitochondrial proteins [17,18]. However, in matured adipocytes, the number of mitochondria is considerably lower compared to preadipocytes. This reduction is due to mitophagy, a specific type of autophagy that degrades mitochondria, which is highly activated during the maturation of adipocytes. Besides decreasing the number of mitochondria as the adipose tissue matures, mitophagy also plays a crucial role in ensuring the proper function of mitochondria in mature adipocytes [19]. Although autophagy is crucial for proper adipocyte function and differentiation, defective regulation associated with obesity results in metabolic abnormalities, leading to the development of MetS [20].

Meanwhile, the exact regulatory mechanisms of autophagy in adipocyte formation remain unclear [21,22]. Thus, the inhibition of autophagy might be expected to accelerate the development of obesity and its associated pathologies. Therefore, it is imperative that the autophagy process in obese patients is thoroughly investigated in order to fully exploit its therapeutic potential in the prevention and treatment of obesity. To date, changes in autophagy, both its increase and decrease, have been shown to be involved in the pathogenesis of various diseases, including cancer, neurological, cardiovascular and metabolic diseases [23].

The many studies show altered (increased or decreased) autophagy in genetically modified or diet-induced animal models of obesity [17,24,25,26,27,28]. Simultaneously, a limited number of studies in obese humans have examined whether there is a possible link between dietary nutrient intake and autophagy-related genes’ expression.

Nutritional interventions that induce autophagy can be used to manipulate metabolism in vivo. A new insight into how dietary components affect autophagy could provide important directions for modifying diets to help prevent and/or treat obesity in humans.

In this review, we discuss the importance of various nutritional interventions as induction regulators of autophagy-related genes in the prevention and/or treatment of obesity in humans.

## 2. Methods

This narrative review was designed and reported in accordance with the guidelines of the preferred reporting items for systematic reviews and meta-analyses (PRISMA). The databases PubMed, Springer, ScienceDirect and Scopus were search using the terms “autophagy-related genes”, “overweight”, “obesity”, “obesity diseases”, “human studies”, and “metabolic syndrome” in combination with “nutrition”, “dietary nutrition intake”, “natural compounds”, “Calorie restriction”, and “Intermittent fasting”. Secondary searches were performed by incorporating the terms “in vitro or in vivo” and “review or clinical trial” alongside the original search terms. Given the vast amount of literature available, only the most pertinent articles were chosen, focusing mainly on the quality of the studies, recent publication dates, and the diversity of mechanisms and models investigated.

The inclusion criteria consisted of (1) English-language full-texts that were peer-reviewed; (2) implementation of a well-defined study design (such as cross-sectional or observational studies), and (3) studies published from 1 January 2010 to 1 July 2024. The exclusion criteria were non-English articles, opinion pieces, scientific dissertations and abstracts. We also excluded studies of short duration (<2 weeks) and studies that focused on intercurrent medical conditions. Two independent reviewers conducted the search and selected the legal acts and list of qualified articles, which we described. Reference lists from all selected articles were also examined for additional relevant studies.

## 3. The Regulatory Mechanism of Autophagy

Autophagy is a highly conservative mechanism of self-digestion, responsible for the removal of damaged proteins and organelles as well as proteins distorted during biosynthesis. The function of the autophagy process is to maintain homeostasis in the body by removing damaged proteins, fatty acids and cellular organelles, which are degraded by lysosomal enzymes [16,23].

Autophagy can be either selective or non-selective, as it has the ability to specifically target certain organelles, such as in the processes of mitophagy or lipophagy. It is induced by nutrient deficiency and stress factors to increase the number of autophagosomes and starts with the formation and extension of a membrane structure called the phagophore, which ultimately develops into an autophagosome—a vesicle with a double membrane. This process involves components from autophagy-related genes and proteins (ATG) [29,30].

**Figure 1:**
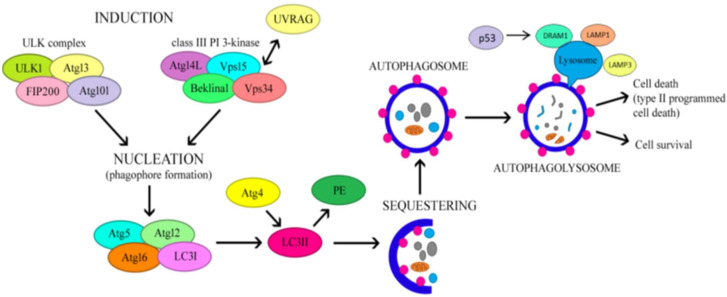
Mechanism regulating autophagy. ATG4—autophagy-related gene 4; ATG5—autophagy-related gene 5; ATG12—autophagy-related gene 12; ATG13—autophagy-related gene 13; ATG14L—autophagy-related gene14L; ATG16—autophagy-related gene 16; ATG101—autophagy-related gene 101; DRAM1—damage-regulated autophagy modulator 1; FIP200—focal adhesion kinase family interacting protein of 200 kD; LAMP1—lysosomal-associated membrane protein 1; LAMP3—lysosomal-associated membrane protein 3; LC3I—microtubule-associated protein light-chain 3 (cytosolic form); LC3II—microtubule-associated protein light-chain 3 (lipophilic form); p53—tumor suppressor p53; PE—phosphatidylethanolamine; ULK1—unc-51 like autophagy activating kinase-1; UVRAG—UV irradiation resistance-associated gene; Vps15—Phosphoinositide 3-Kinase Regulatory Subunit 4; Vps34—vacuolar protein sorting 34.

ATG1 and microtubule-associated protein light chain 3 (LC3) are widely regarded as important autophagy-initiating genes. The conversion of LC3-I to LC3-II leads to the creation of autophagosomes. P62/secretosome (SQSTM) is a protein that binds to ubiquitin and identifies ubiquitinated materials, attaching to autophagosomes through direct interaction with LC3-II [31,32]. Since both LC3-II and p62 are broken down within the autolysosome, their accumulation serves as a strong indicator of dysfunctional autophagy. Lysosome-associated membrane proteins (LAMPs) play a critical role in the fusion of autophagosomes and lysosomes, as well as in the lysosomes’ proteolytic function. When the activities of LAMP1 and LAMP2 are inhibited, the fusion between autophagosomes and lysosomes is also hindered, leading to increased levels of LC3-II and p62 and a decrease in autophagic activity [33,34]—Figure 1.

This mechanism is closely linked to nutritional status. When nutrients are supplied to the body or under the influence of insulin, phosphatidylinositol 3-kinase (PI3K) class I activates mTOR and mTOR complex 1 (mTORC1), thereby inhibiting ATG1 activation. In contrast, the PI3K-beclin1 class III complex is triggered in the presence of nutrient deficiency, which promotes the formation of the ATG12-ATG5-ATG16L and ATG8/LC3 complex and subsequently stimulates autophagosome formation [35].

Following phagophore formation via both the mTOR and PI3K pathways, the phagophore complex and ATG5-ATG12-ATG16 bind to caveolin-1 (CAV-1) and then interact with LC3 to promote autophagosome formation and CAV-1 degradation [24]. During CAV-1 deficiency, increased levels of autophagy-related gene 7 (ATG7), beclin1 and LC3-II were demonstrated, indicating increased autophagy activity and protection against atherosclerosis [35,36].

Components of MetS, including elevated glucose and dyslipidemia [37,38,39], inhibited autophagosome formation through CAV-1 activation [36,37,38,39].

Liver and adipose tissues are abundant in lysosomes and exhibit elevated levels of autophagy triggered by cellular stress. Reactive oxygen species (ROS) can disrupt lysosomal function and hinder autophagy, which obstructs the breakdown of damaged cellular components. Moreover, heightened endoplasmic reticulum (ER) stress can interfere with the acidification of lysosomes, subsequently blocking autophagy in hepatocytes. This leads to hepatotoxicity, cell death and changes in liver function. Obesity, autophagy and metabolic syndrome are mutually dependent.

Studies indicate that human adipose tissue and fat cells contain autophagosomes and show increased expressions of autophagy-related genes, including ATG5, ATG7, ATG12, Beclin1, LC3I(A), LC3II(B), LC3-II and p62 [29,30,31,32,33,34,35,36].

Interestingly, increased autophagy in obese individuals is more pronounced in visceral than subcutaneous adipose tissue, which is also associated with greater metabolic and cardiovascular risk. The rate of fat accumulation and fat cell hypertrophy are significantly associated with autophagy gene expression, and changes in autophagy are accompanied by obesity-associated IR, preceding metabolic and cardiovascular dysfunction [30].

## 4. Autophagy and Obesity Links

Adipose tissue serves as a primary storage site for lipids and is crucial for energy metabolism. The differentiation of adipose tissue involves the significant remodeling of progenitor cells, with one of the key changes being the removal of cytoplasmic elements, particularly mitochondria, during the maturation of adipocytes.

It is likely that autophagosomes help to reorganize cytoplasmic components by transporting membranes within the cell, thereby promoting adipogenesis [20,40,41,42]. Furthermore, studies indicate that autophagy increases the stability of peroxisome proliferator-activated receptor (PPAR) γ, a key regulator of both adipogenesis and adipocyte differentiation. Studies have shown that blocking autophagy reduces PPARγ activity and directly interferes with adipocyte differentiation [43]. PPARγ is the rate-limiting enzyme for adipogenesis and fat accumulation in excessive adipose tissue [44,45]. Thus, PPARγ activation by autophagy may be the mechanism by which autophagy induces obesity, and may become a future target for preventing obesity-associated autophagy during MetS [45,46]. In addition, PPARγ activation during obesity depends on a number of other factors, including polyunsaturated fatty acids (PUFAs) and prostaglandins (e.g., J2 or D2) [47]. Therefore, additional studies are needed to determine exactly whether the activated PPARγ pathway induces or inhibits autophagy during obesity. Adipogenesis is a two-step process in which multipotent adipose tissue-derived mesenchymal stem cells (ASCs) transform into mature adipocytes, which in turn are involved in energy storage in the form of fat. In obesity, an increase in the size and number of these cells leads to adipose tissue proliferation, which is closely related to IR [48]. Autophagy plays a key role in adipogenesis. Adipocyte differentiation is associated with increased levels of autophagy, while the inhibition of autophagy inhibits adipogenesis [49]. The pathogenesis of obesity underlies a significant accumulation of potential autophagic substrates, such as lipid droplets, protein aggregates and damaged mitochondria. Therefore, the inhibition of autophagy can be expected to accelerate the development of obesity and its associated pathologies [50]. However, recent studies indicate that a myriad of intracellular and extracellular factors are involved in the etiogenesis and development of obesity. Therefore, it is imperative that the autophagy process in obese patients is thoroughly investigated in order to fully exploit its therapeutic potential in the prevention and treatment of obesity. To date, changes in autophagy, both its increase and decrease, have been shown to be involved in the pathogenesis of various diseases, including cancer, neurological, cardiovascular and metabolic diseases [51].

### 4.1. Insulin as a Potent Inhibitor of Autophagy

**Figure 2:**
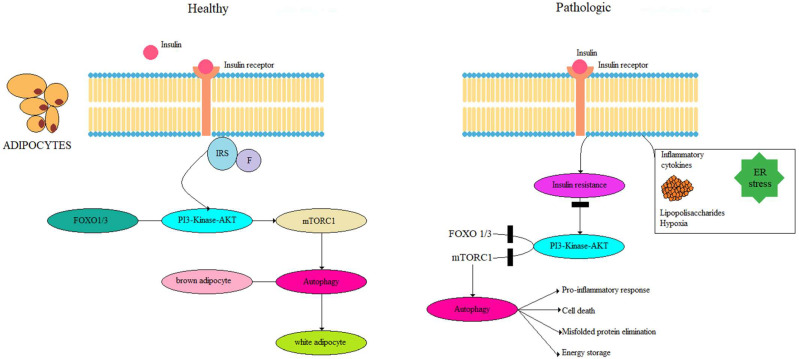
Regulation of insulin signaling pathway in adipocytes in healthy and obese (pathologic) subjects. In the former case, the induction of mTORC1 by insulin results in the inhibition of autophagy. The inhibition of autophagy induces a “browning” phenotype in adipocytes. During obesity, ER stress, hypoxia and inflammation stimulate insulin resistance, resulting in the inhibition of mTORC1, followed by the induction of autophagy. Autophagy improves adipocyte function by eliminating damaged organelles and misfolded proteins and preventing pro-inflammatory responses. In addition, the excessive stimulation of autophagy can increase energy storage by adipocytes and promote cell death.

One potent inhibitor of autophagy is insulin—an anabolic hormone [52]. It does this by activating mTORC1, which in turn suppresses the activity of the FoxO and ULK1 factors. The PI3K-Akt pathway plays a crucial role in the insulin signaling mechanism that leads to this inhibition of autophagy. Specifically, Akt acts to inhibit FoxO1/3 and promotes mTORC1 activity, highlighting a significant connection between insulin signaling and autophagy [53]. However, the targeted deletion of ATG7 causes deleterious white adipose tissue (WAT) differentiation and browning, which ultimately enhances insulin sensitivity, boosts glucose uptake, and increases the β-oxidation of fatty acids [54]. These metabolic changes through the inhibition of autophagy play a key role in IR. It has indeed been shown that a specific deficiency of ATG3 and ATG16L1 in adipocytes caused IR. In particular, the inhibition of autophagy in adipocytes interfered with insulin signaling to Akt in adipose tissue, liver and skeletal muscle. Based on the studies performed to date, the innate inhibition of autophagy impairs adipogenesis and leads to insulin sensitivity, whereas the selective inhibition of this pathway in mature adipocytes results in IR [55,56], as shown in Figure 2.

### 4.2. Role of Autophagy in Fatty Liver Modulation

**Figure 3:**
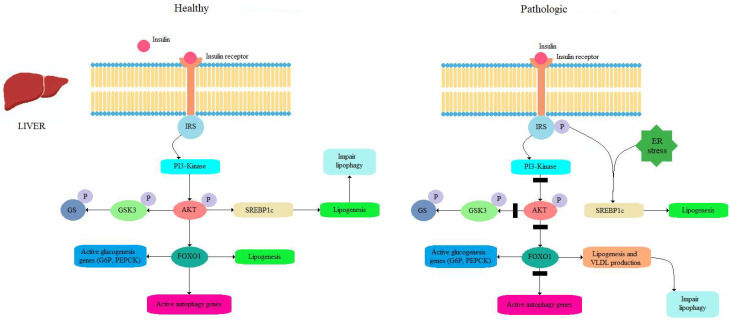
After insulin binds to its receptor in a healthy state, Akt is activated and inhibits GSK3 and FOXO1 in the liver. This leads to increased glycogen synthase activity, decreased hepatic glucose production, and the inhibition of autophagy. In an insulin-resistant state, GSK3 is activated, leading to the inhibition of glycogen synthase. FOXO1 activity increases, resulting in heightened gluconeogenesis, lipid and VLDL synthesis, and increased autophagy. SREBP1c also increases activity, leading to hepatic steatosis. SREBP1c increases its activity through ER stress or through IRS-1.

The action of autophagy in the liver differs significantly from its behavior in adipose tissue during MetS. In obesity, autophagy in hepatocytes is significantly reduced (in contrast, an increase is observed in adipose tissue) because an impaired metabolism is observed in the liver along with deformed mitochondria [57,58]. In contrast to the role of autophagy in adipose tissue, the inhibition of autophagy promotes lipid accumulation in hepatocytes through the lipolysis of lipid droplets accumulated in TG [59]. Furthermore, a constantly positive energy balance favors mTORC1 activity at the expense of AMPK, achieving the consequent inhibition of autophagy [60], as shown in Figure 3.

In a study in mice, it was shown that the long-term use of HFD induced the induction of mTORC1 activity and decreased ATG5 and ATG7 expression in the liver, where autophagy is markedly activated during starvation [61]. Yang et al. [61] showed the lower protein expression of ATG7, beclin1 (ATG6), LC3 and ATG5, and the elevated expression of p62, in livers of obese mice.

Moreover, higher levels of ER and IR stress were observed in these mice due to impaired autophagy activity in hepatocytes. Furthermore, reduced autophagy in the liver was observed in both diet-induced obesity and genetic obesity models, which could be explained by obesity-associated hyperinsulinaemia (insulin inhibits autophagy). Yet, insulin is not the main cause of reduced autophagy in the liver in obese individuals. It is likely that other mechanisms co-exist here. One is the action of calpain 2, a Ca^2+^-dependent protease whose higher levels in hepatocytes reduce autophagy in obese patients [62], and whose inhibition increases autophagy [63]. Another possible mechanism that reduces autophagy in the liver is the transcription factor FoxO, which acts as a key regulator of the Vps34 and ATG12 proteins responsible for the initiation of autophagy [64]. Elevated insulin levels and activated Akt suppress FoxO activity, thereby reducing the rate of autophagy in MetS [65]. Thus, the long-term inhibition of autophagy due to IR and hyperinsulinaemia in MetS can be explained by reduced FoxO activity in hepatocytes [66]. Similarly, the genetic or pharmacological inhibition of autophagy counteracts starvation-induced weight loss while contributing to obesity and T2DM [67]. Therefore, chronic HDF use is thought to alter the intracellular ion balance in hepatocytes, ultimately impeding autophagosome–lysosome fusion [68].

In addition to the altered autophagy in the adipose tissue of obese mice, other tissues such as the hypothalamus and kidney also show lower levels of autophagy [69], suggesting the involvement of systemic factors. Mitochondrial and ER oxidative stress and the accumulation of toxic substances may be responsible for, among other things, inducing IR [70]. In contrast, other authors have reported a relationship between nutrient restriction in obese subjects and an increase in autophagy activity associated with improved insulin sensitivity [71].

Subsequent studies have shown that HFD-induced hepatic steatosis and obesity-related ER stress essentially activate autophagy as a protective mechanism against cellular damage [71]. Autophagy protects hepatocytes from lipotoxicity-related ER stress, as well as from SFA (palmitic acid)-induced apoptosis [72,73], and this may be the reason for the observed induction of autophagy in the early stages of obesity. Nevertheless, studies have shown that the effects of HDF-induced autophagy persist for the first few weeks, with autophagy activity eventually declining due to the ongoing cellular stress that occurs in chronic obesity. Furthermore, a mouse study showed that mRNA and protein levels of beclin1 and LC3 were significantly higher in severely obese mice compared to controls. In contrast, the same obese mice showed significantly reduced LC3-II levels and LC3-II/LC3-I ratios compared to control mice, indicating impaired autophagy [74].

The activation of autophagy facilitates adipocyte differentiation, induces adipogenesis and increases fat accumulation in adipose tissue.

## 5. Autophagy Related Genes in Obese Humans as a Therapeutic Target

The first observations of changes in autophagy in obese patients were reported in 2010 by Öst et al., who, using transmission electron microscopy (TEM), observed an increased number of autophagosomes in adipocytes taken from obese patients with type 2 diabetes [70]. In parallel, an increase in the level of LC3 protein, a major marker of autophagy, was observed, as demonstrated by the use and in the absence of an autophagy inducer (rapamycin) and an inhibitor of lysosomal degradation (chloroquine). Reduced mTORC1 activity further confirmed these findings. Moreover, the observed stimulation of autophagy was associated with the fragmentation of large lipid droplets. In the following years, more studies have been published, demonstrating the overregulation of autophagy markers in AT in patients with obesity and T2D-related obesity [75].

It has been suggested that stimulated autophagy in AT from obese individuals likely plays a role in modulating obesity-induced AT inflammation. This assumption is supported by the findings that the inhibition of autophagy significantly increased the transcriptional expression of pro-inflammatory cytokines in AT samples from obese humans [76,77].

Soussi et al. described a reduction in autophagic flow in adipocytes of subcutaneous tissue in obese subjects [14]. This could be used as a novel biomarker for risk of obesity and age-related chronic disease.

A clinical study by Kovsan et al. [78] involving non-obese, obese and severely obese patients (with and without diabetes) confirmed a possible link between induced autophagy activity and fat accumulation. Authors have shown that ATG 5 and two LC3 genes’ expressions are up-regulated in human subcutaneous and visceral adipose tissue.

Studies by Xu Q et al. [15] performed on adipose tissue (AT) from 17 overweight/obese subjects showed higher expressions of ATG 5, 7 and 12 compared to lean subjects. ATG12 mRNA was positively correlated with BMI and ATG7 mRNA correlated positively with waist/hip ratio, 2 h glucose concentration and insulin. ATG12 conjugates with ATG5 through the concerted action of ATG7 (an E1-like ubiquitin-activating enzyme) and ATG10 (an E2-like ubiquitin-conjugating enzyme), forming the ATG12–ATG5–ATG16L complex, which then participates in LC3–phosphatidylethanolamine conjugation. 

The cross-sectional study of 34 women with metabolically unhealthy obesity, 34 women with metabolically healthy obesity, and 20 healthy non-obese women provided clinical evidence of increased expressions of microRNA-30a and decreased expressions of beclin1 in women with metabolically unhealthy obesity vs. other groups [79]. Beclin1 plays a crucial role in the maintenance of lipid metabolism and mitochondrial function in adipocytes. The miR-30a-5p (main regulator of autophagy) directly interacts with the 3′-UTR of beclin1, and that beclin1 enhances cell autophagy by increasing ATG16 levels [79].

An increase in the transcription factor E2F1 within the adipose tissue of obese patients has been linked to the expression of ATG genes, particularly those that play roles in the later stages of autophagy, such as ATG12, LC3-II, and DRAM1 [80,81]. Adipocytes lacking E2F1 exhibited reduced autophagy activation when exposed to inflammatory cytokines. Notably, the induction of E2F1 in adipose tissue occurs concurrently with inflammatory responses, indicating that the E2F1-mediated activation of autophagy may serve as a defensive strategy against inflammation associated with obesity [82]. These findings suggest a simultaneous relationship between autophagy regulation and inflammation. Various cytokines or adipokines released during episodes of mild inflammation promote autophagy, which is a crucial mechanism for eliminating invading pathogens. Moreover, processes such as hypoxia, inflammation, and ER stress, which occur in adipose tissue during obesity, can stimulate autophagy by inhibiting mTORC1 [77]. In summary, autophagy might act as a protective mechanism against the heightened inflammation linked to obesity, or function as a compensatory response to the surplus of nutrients and damaged organelles found in enlarged adipocytes [83].

Since autophagy may play a key role in preventing or alleviating obesity-related diseases, recent studies have focused on how the autophagy process is altered during obesity, and how autophagic catabolism plays a role in preventing obesity-related comorbidities [58]. Autophagy plays an important protective role against stressors (e.g., lipid accumulation in adipose tissue). For example, the elimination of lipid droplets through the autophagic pathway (known as lipophagy) provides a mechanism for reducing fat and thereby normalizing lipid metabolism in the tissues of obese individuals [40,46,56]. Other studies on human tissues have shown that autophagosomes can accumulate in response to obesity and lipotoxicity in many tissues, including liver and adipose tissue [78].

The liver-specific overexpression of Atg7 or TFEB has also been shown to ameliorate obesity-related ER stress and insulin resistance, confirming the protective role of autophagy in this organ. The pharmacological activation of autophagy, via rapamycin or carbamazepine, also attenuated fat accumulation and liver damage during alcoholic and non-alcoholic fatty liver disease. These results indicate that autophagy plays a protective role against obesity-related liver pathologies. The inhibition of autophagy in adipose tissue has produced some beneficial effects against obesity phenotypes.

Strategies to modulate autophagy are used to treat a variety of conditions, such as the metabolic disorders obesity, MASLD and T2D [68,73].

In human studies [84,85,86], a common observation is that there is heightened autophagy in WAT among individuals who are obese and/or diabetic. Specifically, it was noted that obese individuals exhibited higher levels of mRNA and/or protein for several autophagic markers, including Beclin-1, ATG5, ATG12, ATG7, LC3A and B, LC3-II, and p62, alongside the reduced expression of mTOR in subcutaneous and/or visceral WAT compared to lean individuals.

### Therapeutic Modulation of Autophagy in Obese Humans

Obesity treatment methods that modulate the autophagy process include calorie restriction strategies, calorie restriction mimetics, nutritional interventions, physical exercise, bariatric surgery, and pharmacological drugs. Anti-diabetic drugs that are currently used in clinical practice have modulatory effects on the autophagy process in obese humans:

(a) Metformin—multiple signaling pathways mediate the effect of metformin, which is related to the improvement in the induction of hepatic autophagy and the inhibition of the induction of adipose tissue autophagy [87];
(b) SGLT2 inhibitors—the effects of SGLT2 inhibitors promote the activation of AMPK, SIRT1, SIRT3, and SIRT6, and PGC-1α, decrease the activation of mTOR in diverse tissues under stress, and increase biomarkers of autophagy flux (LAMP-1, Beclin-1) [88];
(c) DPP4 (Dipeptidyl-Peptidase 4) inhibitors promote the autophagy process to decrease triglycerides (TGs), low-density lipoproteins (LDLs) and high-density lipoproteins (HDLs), as well as nitro-oxidative stress, via a mechanism dependent on autophagy modulation [89];
(d) GLP-1 receptor agonists (GLP-1 RA) play a crucial role in regulating autophagic flux, as GLP-1R knockdown suppresses the autophagy induction and mTOR inhibition induced by GLP-1 peptide treatment. Additionally, GLP-1R expression was found to be decreased in certain conditions, such as high-fat diet-induced liver steatosis [90];
(e) GLP/GIP receptor agonist—this novel antidiabetic, anti-obesity drug displayed a protective cardiac effect, preventing cell death, fibrosis and hypertrophy with a potential positive impact on cardiac remodeling. It also plays a crucial role in the autophagic process via the AMPK/mTOR pathway [91].

## 6. Obesity Inhibits or Activates Autophagy?

According to the literature, autophagy can be inhibited or enhanced in obesity. Excess food stress in obesity can stimulate autophagy in certain tissues, while lipotoxicity can interfere with autophagy by inhibiting autophagosome formation. For example, ER stress can induce autophagy through multiple mechanisms, but can inhibit it through others [21,22]. In adipose tissue, obesity increases autophagy, which correlates with the degree of obesity, visceral fat distribution and adipocyte hypertrophy, probably in response to ER stress. It is obvious that autophagy plays different roles in the regulation of metabolic homeostasis in various organs. For example, while autophagy may directly increase during ER stress in pancreatic ß cells or kidney cells, obesity can also cause a decrease in autophagosome degradation, which in turn causes a compensatory increase in the expressions of relevant proteins, resulting in reduced autophagy under these conditions [51,55,57].

Adipose tissue biopsy samples are highly heterogeneous due to the combination of multiple cell types. In addition to adipocytes, it also contains a lining-vascular fraction, which includes inflammatory cells associated with obesity. Therefore, there may be discrepancies in studies, with some studies showing increased autophagy and some showing attenuation. Decreased autophagic flow was inversely correlated with the amount of lipids accumulated in the adipocytes [30,50,66]. The mechanism of impaired autophagy is dependent on DAPK2 (death-associated protein kinase 2), which is one of the most silenced genes in the adipose tissue transcriptome. An impaired systemic metabolism due to excessive food intake along with insufficient energy expenditure, which is associated with obesity, lead to the inhibition of autophagy due to increased mTORC1 signaling. However, this mechanism is more complex. Autophagy can be enhanced or inhibited, depending on changes in the global metabolism of the specific type of organ or tissue under study, as well as experimental conditions or the duration of the disease [31,33]. Importantly, most of these studies have shown changes in autophagy based on changes in the mRNA levels of only a few genes (ATG5, ATG7 i LC3B) [82].

As for the liver, reduced liver BECN1 mRNA levels were observed in patients with simple hepatic steatosis compared to patients with a histologically normal liver [67,69,79]. Reduced levels of BECN1, as well as other proteins, i.e., autophagy-activating kinase Unc-511 (ULK1), p-ULKs555, ATG5, p62/SQSTM1 and BCL2 interacting protein 3 (BNIP3), have been reported in liver samples from MASLD patients compared to control patients with healthy livers, suggesting the impaired initiation of autophagy and autophagosome formation. In conclusion, the available evidence from patients suggests a role for autophagy inhibition in the development and progression of MASLD, but more evidence is needed regarding the stage of autophagy impairment to better understand the pathophysiology of this disease. In obesity, autophagy levels are reduced in hepatocytes. Several mechanisms may be responsible for this decrease. First, an obesity-induced increase in the calcium-dependent protease calpain 2 leads to decreased Atg7 expression, followed by defective autophagy. Acute calpain inhibition is able to restore Atg7 expression. Second, in obesity, the autophagy inhibitor mTOR is overactivated in the liver, probably as a result of increased amino acid concentrations caused by a calorie-rich diet. Finally, although controversial, hyperinsulinemia may also contribute to decreased autophagy in obesity. In obesity, the defect in autophagy and the associated decrease in the rate of lysosome degradation further contribute to the increased ER stress induced by excess nutrients in the inflammatory environment [57,58].

## 7. Nutritional Interventions for Autophagy Modulations in Overweight/Obese Humans

Lifestyle modifications (such as calorie restriction, intermittent fasting, sleep, stress control, various diets, exercise), nutritional interventions and pharmacological modulations of autophagy have been proved to be beneficial in preventing and treating obesity and its complications by improving metabolic health [11]. Nutrition interventions are defined as deliberately planned actions that aim to positively change nutrition-related behaviors, environmental conditions, or an aspect of the health status of an individual, a target group or an entire community [92].

Dietary interventions have emerged as promising therapeutic tools for obesity and metabolic diseases, with limited deleterious side effects. Data from epidemiological, experimental and clinical studies have shown that nutritional strategies are well-known methods for inducing autophagy [31,93,94,95].

### 7.1. Calorie Restriction; Intermittent Fasting

According to a number of studies [96,97,98,99,100,101,102,103,104,105], calorie restriction (CR), an effective, non-genetic and non-drug process (usually 20–40% intake), and intermittent fasting (IF), involving various short- and long-term diet programs with regular cycles between eating and fasting times, stimulate autophagy—Table 1.

CR also alters the level and/or activity of CoA (the sole donor of acetyl groups), acetyltransferases and/or deacetylases, leading to the induction of autophagy through the deacetylation of cellular proteins. The deep activation of autophagy occurs after 72 h of fasting, but 3-day periods without food intake are challenging for individuals. Therefore, various less restrictive dietary models have been developed to help induce autophagy [101].

In a study by Yang et al. [102], serum cortisol, molecular chaperones and autophagy proteins were measured in the skeletal muscles of subjects on CR diets for 3–15 years and in control volunteers. The authors found that CR significantly upregulated a multitude of autophagy genes, including ULK1, ATG101, Beclin-1, APG12, microtubule-associated protein 1 light chain 3 (LC3), GAPRAP/GATE-16, and autophagin-1. At the same time, the expression levels of Beclin-1 and LC3 proteins were significantly higher in the skeletal muscles of the CR volunteers compared to the control group. Chaudhary and co-workers [99] reported that skeletal muscle autophagy may be suppressed in obese woman in response to weight loss—fifty women (51 ± 2 years; BMI 31.8 ± 4.3 kg/m^2^) were randomly assigned to one of two IF protocols (24-h fasting, 3 non-consecutive days per week) and fed at 70% (IF70) or 100% (IF100) of energy requirements for 8 weeks. The vastus lateralis muscle was sampled after 12 and 24 h fasting. The 24 h fast increased mRNA levels of SQSTM1, BECLIN1, SQSTM1 and LAMP2, which were reduced in IF70 after a 12 h overnight fast. The benefits of food and energy restriction in promoting optimal health were supported by Song’s study, which found that IF (60% calorie restriction for 2 days per week or every other day) delays pathological processes through adaptive stress signaling cascades to improve mitochondrial functions, DNA repair and autophagy [103]. Kim and Lee concluded that these findings are consistent with the hypothesis that humans and other animals evolved survival mechanisms in food-deficient environments and developed adaptations to improve both physical and cognitive functions [104].

In a report by Kitada, Kume et al. [105], four overweight male participants were enrolled and treated with 25% CR of their basal energy requirements for 7 weeks, allowing the researchers to demonstrate the effects of human serum taken from CR participants on AMPK and SIRT1 activation and mitochondrial biogenesis in cultured human skeletal muscle cells. AMPK and SIRT1 activation was assessed by the deacetylation of the H_2_O_2_-induced increase in acetylated-p53 expression, and was shown to be significantly increased in human skeletal muscle cells cultured with serum after CR. The authors observed a correlation between SIRT1 gene expression and lower serum levels of insulin, free fatty acids and interleukin 6.

The interplay between Sirtuins and autophagy, both potential longevity-promoting factors, has gained more attention in recent years. SIRT1 is one of the main mammalian sirtuins that is upregulated in response to CR [106]. Other studies [107,108] have shown that SIRT1 is expressed in visceral adipose tissue and is reduced by obesity. Sirtuin 1 (SIRT1) increased basal autophagic activity, and SIRT1 knock-out cells and mice demonstrated unusually high levels of the acetylation of essential autophagic proteins like Autophagy Protein 5 (ATG5), ATG7, and Light Chain 3B (LC3B).

To summarize, the molecular investigations of CR have revealed that low energy levels or the deprivation of essential nutrients (glucose and amino acid) can lead to ATP depletion and increased AMP/ATP ratio. An increased AMP/ATP ratio is the cause AMPK activation. CR decreases mTOR signaling by reducing insulin and IGF levels. Decreased mTOR and activated AMPK efficiently promotes autophagy by directly activating ULK1 Unc-51-like kinase through the phosphorylation of Ser317 and Ser777. The ULK1 complex further activates the BECN1-VPS34-ATG14L-p150 complex through the phosphorylation of Beclin 1 (BECN1). The activation of the BECN1 complex leads to the generation of phosphatidylinositol-3-phosphate (PI3P), which is crucial for the nucleation of autophagic vesicles [100,105]. The serine/threonine protein kinase ULK1 (unc-51-like kinase 1) operates within a complex that includes at least three protein partners, as follows: FIP200 (focal adhesion kinase family interacting protein of 200 kDa), ATG13 (autophagy-related protein 13), and ATG10. This complex is crucial for regulating the formation of autophagophores, which are the precursors to autophagosomes. Additionally, ULK1 serves as both a downstream effector and a negative regulator of the mammalian target of rapamycin complex 1 (mTORC1) [109,110].

One common form of IF is time-restricted eating (TRE), which confines caloric intake to a 6 to 10 h period, without altering the overall quantity or quality of the diet. Overweight adults (six females and three males, aged 65 and older) were instructed to fast for about 16 h a day for four weeks, with a daily target fasting duration of 14 to 18 h. During the designated 16 h fasting period, participants refrained from consuming any calories. They faced no limits on the amount or types of food they could consume during the 8 h eating window, and they were allowed to choose a timeframe that suited their lifestyle best. Blood samples were taken from all participants before and after the TRE regimen in the morning, and the expression levels of 2083 human microRNAs were measured using an HTG molecular whole-transcriptome miRNA assay. Ultimately, 14 miRNAs showed differential expressions before and after the TRE regimen. Importantly, the downregulated miRNA targets indicated an increased expression of transcripts such as PTEN, TSC1, and ULK1 [111]. It has been suggested that the suppression of mTOR signaling and increased autophagy contribute to many of the beneficial adaptations observed in TRE protocols in clinical populations.

The beneficial effects on autophagy of both CR and IF appear to be associated with increases in fat mobilization, oxidation, metabolic flexibility, insulin sensitivity and redox imbalance, along with a reduction in systemic inflammation, cardiovascular risks and body weight.

Many nutrients with significant health/translational properties (nutraceuticals) may affect the chronic disease risk through various mechanisms that include the activation or inhibition of autophagy [31,112,113,114,115]. These include, for example, amino acids (i.e., leucine), fatty acids (i.e., omega 3 polyunsaturated fatty acids), vitamins (carotenoids and retinoids, ascorbic acid, calciferol, tocopherols, and tocotrienols), coenzyme Q10, bioactive compounds (i.e., mainly polyphenols like curcumin, caffeine, EGCG, resveratrol, allicin), minerals (zinc or iron), ergothioneine, lipoic acid, N acetylcysteine and spermidine.

According to a number of studies [31,116,117,118], the Mediterranean diet (Med Diet) also has significant effects on the regulation of autophagy.

### 7.2. Mediterranean Diet (Met Diet)

In 2010, UNESCO acknowledged the Mediterranean diet as an Intangible Cultural Heritage of Humanity and developed the food pyramid model in order to communicate the “Med Diet” model to people and health professionals [116].

The Mediterranean diet is characterized by a high intake of plants and is rich in dietary fiber, vitamins, polyunsaturated fatty acids, oligoelements, polyphenols, and others [117,118,119]. One of the dietary ingredients common in plant-based diets is polyphenols, which are particularly abundant in fruits, vegetables, whole grains, and legumes, but also in cocoa, tea, coffee, and red wine [118,119].

Polyphenols are classified as flavonoids, with seven described subclasses (flavonols, flavones, flavanones, flavanonols, flavanols, anthocyanidins, and isoflavones) and non-flavonoid molecules (phenolic acids, hydroxycinnamic acids, lignans, stilbenes, and tannins) [120,121]. Flavonoids are polyphenolic secondary metabolites that are commonly found in most plants. These compounds can occur as glycosides or aglycones. Flavonoids exhibit a broad spectrum of biological activities, such as neuroprotective, anti-inflammatory, antibacterial, hepatoprotective, anti-mutagenic, anticancer, cardiovascular protective, antifungal, antiviral, and anti-allergic effects [122].

The high content of protective phenolic compounds in the Med Diet’s ingredients, especially those present in vegetables and fruits, may also help explain their multiple benefits [123]. Some well-known polyphenols include resveratrol, quercetin, curcumin, epigallocatechin gallate, catechin, hesperetin, cyanidin, procyanidin, caffeic acid, and genistein [124]. A study by Osorio-Conles and co-workers [125] evaluated the short-term effects of a dietary intervention based on the Mediterranean diet (Med Diet) supplemented with almonds (MDSA) on the main features of obesity-related white adipose tissue (WAT) dysfunction. A total of 38 obese women (aged 18–68 years, with a BMI of 40–50 kg/m^2^) were randomly assigned to a 3-month intervention with MDSA (19 women) vs. maintaining their usual diet (17 women). Biopsies of subcutaneous (SAT) and visceral adipose tissue (VAT) were obtained before and after the dietary intervention. The expression of angiogenesis-related genes PDGFRB, VEGFA, VEGFR1 and VEGFR2 was significantly increased after MDSA intervention compared to controls. In VAT, the expression of genes associated with adipogenesis, angiogenesis, autophagy and fatty acid usage was increased. Among other things, the authors found increased expressions of autophagy-related ATG 7 and ATG12 in VAT from the MDSA group, while ATG5 showed a non-significant trend (*p* = 0.054).

### 7.3. Dietary Polyphenols

Dietary polyphenols (e.g., Resveratrol, Epigallocatechin-3-gallate (EGCG), curcumin, quercetin, chlorogenic acid) have beneficial effects on fat mass in humans. Fruits, whole grains, vegetables, and other types of foods and beverages, such as tea, chocolate, and wine, are rich sources of these [126,127].

Autophagic pathways, including cAMP, AMPK, MAPK, AKT, SIRT1, PI3K, Nrf2/HO-1, PINK1/Parkin, PPARδ, and miRNAs, have been implicated in the amelioration of glucolipid metabolic diseases by plant polyphenols [126,127,128].

The effect of green tea consumption in the forms of EGCG or green tea polyphenols with or without caffeine on obesity-related parameters has been reported in many studies, but not all studies have shown positive results for obesity-related parameters [120,121,126,129].

Resveratrol (RES) is a well-known polyphenolic compound found in various plants. Park et al. [130] suggested that resveratrol stimulates autophagy through convergent modalities that include the activation of the AMPK–SIRT1–PGC-1α axis and the inhibition of the mTOR-ULK1 pathway. According Widlund et al. [131], RES plays a role in modulating mTOR pathway proteins, although further studies are still needed to fully understand this interaction.

In a placebo-controlled, double-blind cross-over study [132], 11 healthy obese patients (52.5 ± 2.1 years) received placebo followed by 150 mg of resveratrol once daily for 30 days after a 4-week break. After muscle biopsy, the authors also found increased AMPK phosphorylation and SIRT-1 and PGC-1α expression. Konings E, Timmers S et al. [133] indicated that in the obese men analyzed previously, it reduced the size of subcutaneous adipocyte in the abdominal region and enhanced and improved adipogenesis, presumably through modulating gene expression. At the same time, RES supplementation induced, among other things, the expression of TFEB (transcriptional factor EB) in the subcutaneous AT. TFEB controls many key steps in the autophagy pathway [134]. Moskot M, Montefusco S et al. [135] discovered a regulatory network linking the phytoestrogen genistein-mediated control of EB transcription factor (TFEB) gene expression, TFEB nuclear translocation, and the activation of TFEB-dependent lysosome biogenesis to lysosomal metabolism.

In a randomized, placebo-controlled study by Most et al. [127], 25 (10 women) overweight and obese subjects received a combination of the polyphenols epigallocatechin gallate (EGCG) and resveratrol (RES) (282 mg/d, 80 mg/d, EGCG+RES, respectively, n = 11) or placebo (PLA, n = 14) for 12 weeks. Subcutaneous adipose tissue (SAT) biopsies were taken to assess adipocyte morphology and perform a microarray analysis. EGCG+RES supplementation had no significant effect on mean adipocyte size or area in abdominal subcutaneous adipose tissue, and showed that it can induce the suppression of gene sets related to adipocyte turnover (adipogenesis and apoptosis/autophagy), inflammation and the immune system in AT in overweight and obese subjects. It increased the expressions of genes ATP6V1A, ATP6V1H, CD68, HSL/LIPE, LAMP2, PI4K2A, UCP2, and GAPDH.

Forty-eight healthy participants aged 55–65 years with a BMI < 29.9 kg/m^2^ were randomly assigned to 30 days of resveratrol (500 mg/day) or calorie restriction (1000 cal/day) in the study by Mansur and colleagues [136]. Plasma SIRT1 levels and gene expression were studied by real-time PCR. Resveratrol and caloric restriction increased serum SIRT1 levels from 1.06 ± 0.71 to 5.75 ± 2.98 ng/mL and from 1.65 ± 1.81 to 5.80 ± 2.23 ng/mL, respectively. The potential role of resveratrol in the treatment and prevention of obesity requires further research, and more standardized clinical trial designs are needed to adequately investigate the benefits of this polyphenol [137,138].

### 7.4. Dietary Fatty Acids

It has been observed that dietary interventions can affect autophagy in subcutaneous visceral adipose tissue in obese individuals. Studies indicate that this effect depends on the specific types of fat, protein and carbohydrate consumed [84]. A high-fat diet is one of the factors contributing to obesity, and the fatty acid profile of the diet plays a significant role in the development of obesity and the involvement of autophagy in this process [139]. Studies have shown that saturated fatty acids (SFAs) are more likely to promote obesity compared to monounsaturated fatty acids (MUFAs) and polyunsaturated fatty acids (PUFAs) [140]. As the most common monounsaturated fatty acid (MUFA) in the daily diet, oleic acid can induce autophagy, which is responsible for regulating lipid metabolism in hepatocytes. ω-3 and ω-6 polyunsaturated fatty acids (PUFAs) are essential for normal physiology and metabolism, and play a role in the occurrence and development of many diseases [141]. O’Rourke’s data showed that ω-6 PUFA supplementation activates autophagy in human epithelial cells [142].

According to Ciesielska K and Gajewska M [143], saturated fatty acids (SFAs) trigger autophagy, which appears to be closely related to the activation of the diabetogenic stress kinase JNK1 and the increased endoplasmic reticulum (ER) stress observed in hypertrophic adipocytes. Polyunsaturated fatty acids (PUFAs), on the other hand, have a protective effect, as they can alleviate SFA-induced mitochondrial dysfunction, reduce oxidative stress, and regulate the inflammatory response in adipose tissue.

Yang B and colleagues [144] found that omega-6 PUFAs (such as linoleic acid) stimulate both autophagy and antioxidant processes in a synergistic feedback loop through TOR-dependent and TOR-independent signaling pathways—through the adenosine monophosphate-activated protein kinase (AMPK) and the AMPK-target of rapamycin (TOR) signaling pathways.

Camargo et al. [145] conducted an RCT involving 39 obese participants with metabolic syndrome, who followed one of four diets for 12 weeks. The diets included a high-saturated fatty acid diet (HSFA), a high-monounsaturated fatty acid diet (HMUFA), and two low-fat, high-complex-carbohydrate diets—one supplemented with long-chain *n*-3 polyunsaturated fatty acids (LFHCC *n*-3) and the other with a placebo (LFHCC). Following the dietary intervention, subcutaneous adipose tissue samples were taken from the abdominal area. The researchers found that long-term adherence to the HMUFA diet significantly increased the expressions of autophagy-related genes (BECN1 and ATG7). Additionally, the LFHCC and LFHCC *n*-3 diets resulted in the increased expression of the apoptosis-related gene CASP3. The expression levels of other autophagy markers examined (LC3, LAMP2, and ULK1) also tended to increase after participants consumed the LFHCC *n*-3 diet. The number of autophagy-related genes tended to increase after the long-term use of the LFHCC *n*-3 diet, consistent with previous descriptions of apoptosis and autophagy that had been related. The authors concluded that enhanced autophagy may contribute to the maintenance of adipose tissue homeostasis.

The studies described above indicate that PUFAs may be involved in the regulation of autophagy directly, but also indirectly via their small bioactive lipid mediators.

### 7.5. Diet Modifications

In the European multicenter NUGENOB (Nutrient-Gene Interaction in Human Obesity) study involving 648 participants [146,147], subjects were randomly assigned to a 10-week dietary intervention featuring two types of hypo-energetic diets: a low-fat, high-carbohydrate diet (LF) and a moderate-fat, low-carbohydrate diet (MF). Both diets were designed to provide 600 kcal/day less than the individuals’ estimated energy requirements. The LF diet provided 20–25% fat and 60–65% carbohydrates, while the MF diet comprised 40–45% fat and 40–45% carbohydrates. Both diets contained 15% of total energy from protein. Gene expression in adipose tissue was assessed for two groups of 47 obese participants from each dietary regimen, both before and after the 10-week period. The results indicate that the expression levels of five specific genes—FABP4, SIRT3, NR3C1, GABARAPL2, and FNTA—were 15–65% higher in the MF group compared to the LF group. This study shows that energy restriction had a more significant effect on the gene expression changes in human adipose tissue than the macronutrient composition of the diets. Additionally, the regulation of a subset of genes sensitive to macronutrient changes may play a role in adipose tissue function and metabolic response.

### 7.6. Protein Intake

Protein intake is one of the strongest dietary regulators of circulating levels of IGF-1, a potent growth factor that activates the Akt/mTOR pathway [147]. According Liu Ch, Ji L et al. [113], the regulation of autophagy by amino acids is still a burgeoning field of research. In particular, functional amino acids (FAAs) (e.g., arginine, leucine, glutamine, and methionine) are involved in protein synthesis and homeostasis, and regulate autophagy through the sensor-mediated activation of mTORC1 kinase. In a clinical study by Xu C. et al. [148], 19 morbidly obese participants (BMI approximately 45 kg/m^2^) undergoing bariatric surgery were analyzed in two hypocaloric (1500–1600 kcal/day) dietary groups, low protein (10E% protein) and high protein (30E% protein), for three weeks prior to surgery. Serum levels of intrahepatic lipids (IHL) and fibroblast growth factor 21 (FGF21) were measured before and after the dietary intervention. Autophagy flux, histology, mitochondrial activity and gene expression analyses were performed in liver samples collected during surgery. From dynamic analyses of autophagy flux after 3 weeks of intervention, this study confirmed that the LP group displayed significantly elevated autophagy flux and FGF21 levels in the liver and circulation compared to HP, but the HP diet more was more effective in reducing intrahepatic fat. The expression of autophagy-related genes (LC3A, LC3B and Atg5) was positively correlated with ER-stress-related genes (BiP, XBP1s, XBP1, ATF4 and DDIT3).

**Table 1:** Modulation of autophagy by dietary strategy in overweight/obese humans.

Dietary Strategies	Model of Obesity	Parameter Studies	Effect on Autophagy	References
Calorie restriction or Intermittent fasting	Skeletal muscle of body fat-matched endurance athletes; skeletal muscle of obese women; obese humans (subcutaneous, white adipose tissue)	Decreased mTOR signaling through reducing insulin and IGF-1 levels and increased the AMP/ATP ratio, which leads to the activation of AMPK as well as several other products involved in the stimulation of this process (ATG 5, ATG6, ATG7, ATG8, LC3-II, Beclin1, p62, SIRT1, LAMP2, ULK1 and ATG101)	Enhanced	[96,97,98,99,100,101,102,103,104]
Calorie restriction 25% for 7 weeks	Peripheral blood mononuclear cells (PBMNCs) of overweight male	Activated AMPK and SIRT1	Enhanced	[105]
Mediterranean diet (MD) vs. Mediterranean diet with almonds (MDSA)	Obese humans (subcutaneous, white adipose tissue)	Elevated levels of autophagy-related proteins ATG7 and ATG12 were observed in the VAT of the MDSA and ATG5 showed a non-significant trend	Enhanced	[125]
Epigallocatechin-3-gallate + resveratrol (280 mg + 80 mg/d) vs. placebo—12 weeks	Obese humans (subcutaneous, white adipose tissue)	Activated gene expressions of ATP6V1A, ATP6V1H, CD68, HSL/LIPE, LAMP2, PI4K2A, UCP2, GAPDH	Enhanced	[128]
Resveratrol 150 mg once daily for 30 days	Obese men (skeletal muscle)	Activated AMPK, increased SIRT1 and PGC-1α protein levels	Enhanced	[132]
Resveratrol 150 mg once daily for 30 days	Obese men (skeletal muscle)	Activated TFEB (transcriptional factor EB) expression; inhibited mTOR activity	Enhanced	[133]
Resveratrol (500 mg/d) vs. Calorie restriction (1000 kcal/d)	Overweight humans (blood)	Resveratrol and caloric restriction significantly increased serum concentrations of SIRT1 proteins	Enhanced	[136]
4 diets followed a period of 12 weeks: a diet high in saturated fatty acids (HSFA), a diet rich in monounsaturated fatty acids (HMUFA), and two low-fat, high-complex-carbohydrate diets, one of which was supplemented with long-chain *n*-3 polyunsaturated fatty acids (LFHCC *n*-3) while the other received a placebo (LFHCC)	Obese humans (subcutaneous); white adipose tissue	HMUFA diet significantly increased expression of *BECN1* and *ATG7*. LFHCC and LFHCC *n*-3 diets led to an increase in the expression of the apoptosis-related gene *CASP3*. Additionally, the LFHCC *n*-3 diet showed a tendency to increase the expressions of other autophagy markers, such as LC3, LAMP2, and ULK1.	Enhanced	[145]
Low-fat, high-carbohydrate diet (LF) vs. moderate-fat, low-carbohydrate diet (MF) for 10 weeks	Obese humans (subcutaneous); white adipose tissue	Expressions of FABP4, SIRT3, NR3C1, GABARAPL2, and FNTA genes were 15–65% higher in the MF than the LF	Enhanced in MF diet vs. LF	[146,147]
Hypocaloric diet (1500–1600 kcal/day) and low protein LP (10%) vs. hypocaloric (1500–1600 kcal/day) and high protein (30E%) for 3 weeks prior to bariatric surgery	Liver sample collected during surgery	Significantly elevated autophagy flux and FGF21 levels in the livers of patients in the LP diet versus HP	Enhanced in LP diet vs. HP	[148]

## 8. Limitations

The current review has some limitations. First, most experimental studies on this topic have been conducted on yeast, cell lines or animal models. Second, most human studies mainly evaluate anthropometric measurements and selected biochemical indicators before, during and after the designed diet. They do not, for example, address changes in autophagy gene expression and other processes (e.g., apoptosis) in adipose tissue (visceral or subcutaneous), which is mainly due to the invasiveness of the procedure of collecting adipose tissue. So, many questions about nutrient bioavailability, optimal dosage and overall efficacy remain unanswered. Third, at the same time, a limited number of studies in obese people have examined whether there is a possible link between dietary nutrient intake and the expression of autophagy-related genes. There are also few clinical studies with a sufficiently large number of participants (patients, volunteers) emphasizing the importance of dietary and lifestyle strategies in health maintenance and secondary and tertiary prevention. Fourth, clinical trials of autophagy-inducing nutrients are strongly encouraged, while emphasizing the dose, duration and possible synergistic effects of various compounds.

## 9. Conclusions

Autophagy is crucial for the proper functioning and differentiation of adipocytes, and its defective regulation associated with obesity results in metabolic abnormalities, leading to the development of metabolic syndrome. The increased expression of autophagy-related genes correlates with the degree of visceral fat mass obesity, and adipocyte hypertrophy and autophagy in adipose tissue are associated with impaired glucose tolerance in a manner independent of BMI and insulin. Many nutrients with significant health/translational properties (nutraceuticals) can affect chronic disease risk through various mechanisms that include the activation or inhibition of autophagy. The effects of nutritional intervention on the regulation of autophagy in obesity and its comorbidities are not yet clear, especially in obese individuals, and many questions about nutrient bioavailability, optimal dosage and overall efficacy remain unanswered.
